# Assessment of Neurodevelopment in Infants With and Without Exposure to Asymptomatic or Mild Maternal SARS-CoV-2 Infection During Pregnancy

**DOI:** 10.1001/jamanetworkopen.2023.7396

**Published:** 2023-04-10

**Authors:** Morgan R. Firestein, Lauren C. Shuffrey, Yunzhe Hu, Margaret Kyle, Maha Hussain, Catherine Bianco, Violet Hott, Sabrina P. Hyman, Mia Kyler, Cynthia Rodriguez, Melanie Tejeda Romero, Helen Tzul Lopez, Carmela Alcántara, Dima Amso, Judy Austin, Jennifer M. Bain, Jennifer Barbosa, Ashley N. Battarbee, Ann Bruno, Sharon Ettinger, Pam Factor-Litvak, Suzanne Gilboa, Sylvie Goldman, Cynthia Gyamfi-Bannerman, Panagiotis Maniatis, Rachel Marsh, Tyler Morrill, Mirella Mourad, Rebecca Muhle, Gabriella Newes-Adeyi, Kimberly G. Noble, Kally C. O’Reilly, Anna A. Penn, Lawrence Reichle, Ayesha Sania, Vera Semenova, Wendy G. Silver, Grace Smotrich, Alan T. Tita, Nim Tottenham, Michael Varner, Martha G. Welch, Noelia Zork, Donna Garey, William P. Fifer, Melissa S. Stockwell, Catherine Monk, Fatimah Dawood, Dani Dumitriu

**Affiliations:** 1 Department of Psychiatry, Columbia University Irving Medical Center, New York, New York; 2 Department of Pediatrics, Columbia University Irving Medical Center, New York, New York; 3 Department of Psychology, Columbia University, New York, New York; 4 School of Social Work, Columbia University, New York, New York; 5 Heilbrunn Department of Population and Family Health, Columbia University Irving Medical Center, New York, New York; 6 Department of Neurology, Division of Child Neurology, Columbia University Irving Medical Center, New York, New York; 7 Department of Obstetrics and Gynecology, University of Alabama at Birmingham, Birmingham; 8 Department of Obstetrics and Gynecology, University of Utah Health Sciences Center, Salt Lake City, Utah; 9 Department of Obstetrics and Gynecology, Columbia University Irving Medical Center, New York, New York; 10 Department of Epidemiology, Mailman School of Public Health, Columbia University Irving Medical Center, New York, New York; 11 COVID-19 Response, Centers for Disease Control and Prevention, Atlanta, Georgia; 12 Department of Obstetrics, Gynecology and Reproductive Sciences, University of California San Diego, La Jolla, California; 13 New York State Psychiatric Institute, New York, New York; 14 Abt Associates, Rockville, Maryland; 15 Department of Behavioral Sciences, Teachers College, Columbia University, New York, New York; 16 Department of Pathology and Cell Biology, Columbia University Irving Medical Center, New York, New York; 17 Department of Pediatrics, Creighton University School of Medicine, Phoenix Regional Campus, Phoenix, Arizona

## Abstract

**Importance:**

Associations between prenatal SARS-CoV-2 exposure and neurodevelopmental outcomes have substantial public health relevance. A previous study found no association between prenatal SARS-CoV-2 infection and parent-reported infant neurodevelopmental outcomes, but standardized observational assessments are needed to confirm this finding.

**Objective:**

To assess whether mild or asymptomatic maternal SARS-CoV-2 infection vs no infection during pregnancy is associated with infant neurodevelopmental differences at ages 5 to 11 months.

**Design, Setting, and Participants:**

This cohort study included infants of mothers from a single-site prospective cross-sectional study (COVID-19 Mother Baby Outcomes [COMBO] Initiative) of mother-infant dyads and a multisite prospective cohort study (Epidemiology of Severe Acute Respiratory Syndrome Coronavirus 2 in Pregnancy and Infancy [ESPI]) of pregnant individuals. A subset of ESPI participants was subsequently enrolled in the ESPI COMBO substudy. Participants in the ongoing COMBO study were enrolled beginning on May 26, 2020; participants in the ESPI study were enrolled from May 7 to November 3, 2021; and participants in the ESPI COMBO substudy were enrolled from August 2020 to March 2021. For the current analysis, infant neurodevelopment was assessed between March 2021 and June 2022. A total of 407 infants born to 403 mothers were enrolled (204 from Columbia University Irving Medical Center in New York, New York; 167 from the University of Utah in Salt Lake City; and 36 from the University of Alabama in Birmingham). Mothers of unexposed infants were approached for participation based on similar infant gestational age at birth, date of birth, sex, and mode of delivery to exposed infants.

**Exposures:**

Maternal symptomatic or asymptomatic SARS-CoV-2 infection.

**Main Outcomes and Measures:**

Infant neurodevelopment was assessed using the Developmental Assessment of Young Children, second edition (DAYC-2), adapted for telehealth assessment. The primary outcome was age-adjusted standard scores on 5 DAYC-2 subdomains: cognitive, gross motor, fine motor, expressive language, and receptive language.

**Results:**

Among 403 mothers, the mean (SD) maternal age at delivery was 32.1 (5.4) years; most mothers were of White race (240 [59.6%]) and non-Hispanic ethnicity (253 [62.8%]). Among 407 infants, 367 (90.2%) were born full term and 212 (52.1%) were male. Overall, 258 infants (63.4%) had no documented prenatal exposure to SARS-CoV-2 infection, 112 (27.5%) had confirmed prenatal exposure, and 37 (9.1%) had exposure before pregnancy or at an indeterminate time. In adjusted models, maternal SARS-CoV-2 infection during pregnancy was not associated with differences in cognitive (β = 0.31; 95% CI, −2.97 to 3.58), gross motor (β = 0.82; 95% CI, −1.34 to 2.99), fine motor (β = 0.36; 95% CI, −0.74 to 1.47), expressive language (β = −1.00; 95% CI, −4.02 to 2.02), or receptive language (β = 0.45; 95% CI, −2.15 to 3.04) DAYC-2 subdomain scores. Trimester of exposure and maternal symptom status were not associated with DAYC-2 subdomain scores.

**Conclusions and Relevance:**

In this study, results of a novel telehealth-adapted observational neurodevelopmental assessment extended a previous finding of no association between prenatal exposure to maternal SARS-CoV-2 infection and infant neurodevelopment. Given the widespread and continued high prevalence of COVID-19, these data offer information that may be helpful for pregnant individuals who experience asymptomatic or mild SARS-CoV-2 infections.

## Key Points

**Question:**

Is asymptomatic or mild maternal SARS-CoV-2 infection compared with no infection during pregnancy associated with observable infant neurodevelopmental differences at ages 5 to 11 months?

**Findings:**

In this cohort study involving a geographically diverse cohort of 407 infants born to 403 mothers, no association was found between mild or asymptomatic maternal SARS-CoV-2 infection during pregnancy and infant cognition, language, or motor development as assessed by a novel telehealth-adapted version of the Developmental Assessment of Young Children, second edition.

**Meaning:**

Given the continued high prevalence of SARS-CoV-2 infection globally, these data offer information regarding infant neurodevelopment that may be helpful for pregnant individuals with asymptomatic or mild SARS-CoV-2 infections.

## Teaser

This cohort study assesses the association between prenatal exposure to mild or asymptomatic maternal SARS-CoV-2 infection vs nonexposure and differences in neurodevelopmental outcomes among infants aged 5 to 11 months.

## Introduction

Since the onset of the COVID-19 pandemic, researchers and clinicians have considered the short- and long-term consequences of maternal prenatal SARS-CoV-2 infections for child development.^1,2,3,4,5^ Associations between neurobehavioral outcomes and in utero exposures to viruses such as Zika, influenza, and herpes simplex have been widely studied. However, data remain limited about children’s neurodevelopmental outcomes after prenatal exposure to maternal infection with SARS-CoV-2 and other novel coronaviruses, such as severe acute respiratory syndrome and Middle East respiratory syndrome coronaviruses.^6,7^

Given the high burden of SARS-CoV-2 infections among pregnant individuals,^8,9^ understanding the association between prenatal SARS-CoV-2 exposure and infant neurodevelopment remains important to assessing and mitigating the long-term consequences of the COVID-19 pandemic for children’s health. Few documented cases of vertical transmission of SARS-CoV-2 have been reported, suggesting robust fetal protection against infection.^10,11,12,13^ However, neurodevelopment can be altered through other mechanisms, such as maternal immune activation,^14^ necessitating the longitudinal follow-up of children born to individuals who had COVID-19 during pregnancy.

Although limited, data on infants prenatally exposed to a maternal SARS-CoV-2 infection have not suggested an association with adverse neurodevelopmental outcomes. The ongoing prospective COVID-19 Mother Baby Outcomes (COMBO) Initiative^15^ previously reported no neurodevelopmental differences at age 6 months in infants with and without exposure to maternal SARS-CoV-2 infection during pregnancy on any of the 5 subdomains of the parent-reported Ages & Stages Questionnaires, third edition (ASQ-3),^16^ in a cohort from New York City. Similar null results on the ASQ-3 among infants with prenatal exposure to maternal SARS-CoV-2 infection have been reported from other geographical regions, including Kuwait^17^ and China^18^; however, it should be noted that both the COMBO Initiative^16^ and other research groups^19,20^ have found slightly lower parent-reported developmental scores in infants born during the pandemic compared with those born before the pandemic. To date, the cumulative available data have relied on parent-reported measures such as the ASQ-3, which has only moderate sensitivity and specificity for estimating actual neurodevelopmental delays.^21^ Furthermore, parent-reported measures of infant development might be at greater risk of biased perception during a global pandemic due to parental stress and reduced parental exposure to other children and typical infant developmental trajectories. It is therefore important to use standardized observational measures to assess associations between maternal SARS-CoV-2 infection status during pregnancy and neurodevelopmental outcomes.

As part of the continued efforts of the COMBO Initiative,^15^ we conducted a standardized observer-based assessment through remote telehealth visits of infants with and without prenatal exposure to maternal SARS-CoV-2 infection. The aim of this study was to examine whether mild or asymptomatic maternal SARS-CoV-2 infection compared with no infection during pregnancy is associated with neurodevelopmental differences in infants aged 5 to 11 months. In partnership with the Centers for Disease Control and Prevention (CDC), we expanded the COMBO cohort to include mother-infant dyads from 3 distinct geographic regions across the US, enhancing the generalizability of our findings.

## Methods

### Study Design and Participants

**Figure 1.**
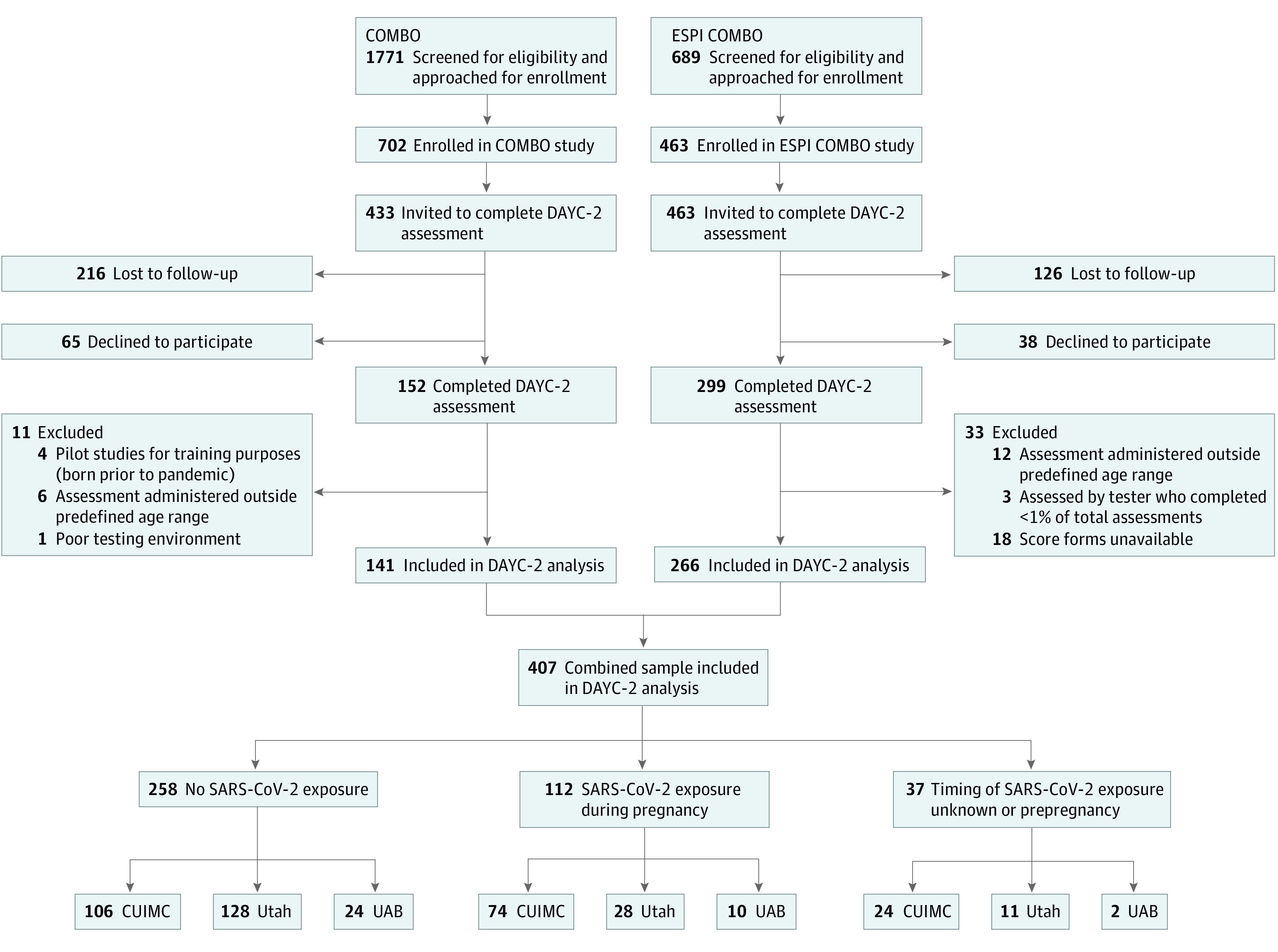
Cohort Flowchart COMBO indicates COVID-19 Mother Baby Outcomes; CUIMC, Columbia University Irving Medical Center; DAYC-2, Developmental Assessment of Young Children, second edition; ESPI COMBO, Epidemiology of Severe Acute Respiratory Syndrome Coronavirus 2 in Pregnancy and Infancy plus COMBO substudy; UAB, University of Alabama; and Utah, University of Utah.

Pregnant individuals and mother-infant dyads were enrolled in 1 of 2 parallel studies: the COMBO Initiative single-site prospective cross-sectional study of mother-infant dyads^15^ or the CDC Epidemiology of SARS-CoV-2 in Pregnancy and Infancy (ESPI) Network multisite prospective cohort study of pregnant women^9^ (Figure 1). A subset of ESPI participants was subsequently enrolled in the ESPI COMBO substudy. Participants in the ongoing COMBO study were enrolled beginning on May 26, 2020; participants in the ESPI study were enrolled from May 7 to November 3, 2021; and participants in the ESPI COMBO substudy were enrolled from August 2020 to March 2021 (only month and year are provided because the exact dates could lead to participant identification). For the current analysis, infant neurodevelopment was assessed between March 2021 and June 2022. All study procedures for the COMBO study were approved by the Columbia University Irving Medical Center (CUIMC) Institutional Review Board (IRB). For the ESPI COMBO substudy, the CDC, the University of Utah, the University of Alabama, and Abt Associates IRBs relied on the review of the CUIMC IRB (per US regulations on the protection of human participants [45 CFR §46^22^] and IRBs [21 CFR §56^23^]). Written informed consent for study procedures, including the use of video and photographic footage, was obtained from all participants, and participants received financial compensation for their time. This study followed the Strengthening the Reporting of Observational Studies in Epidemiology (STROBE) guideline for cohort studies.

The ongoing COMBO Initiative seeks to understand the health and well-being of mothers and infants during the pandemic through both cross-sectional and prospective cohort study designs with longitudinal follow-up of mothers and infants from CUIMC in New York, New York. The completed ESPI COMBO substudy was a prospective cohort study with longitudinal follow-up of participants enrolled in ESPI who completed additional assessments taken from the COMBO protocol between delivery and 6 months post partum; these participants were enrolled from CUIMC, the University of Alabama in Birmingham, and the University of Utah in Salt Lake City. Mothers of unexposed infants were approached for participation based on similar infant gestational age at birth, date of birth, sex, and mode of delivery to exposed infants. Detailed descriptions of recruitment, enrollment, and study procedures for each cohort are described in Table 1 and eMethods 1 in Supplement 1. Data were available from 407 infants (141 born between April 2020 and May 2021 who were enrolled in the COMBO study plus 266 infants born between January and September 2021 who were enrolled in the ESPI COMBO substudy), including 9 twins (8 belonging to a twin pair) and, therefore, 403 mothers.

**Table 1.** COMBO and ESPI COMBO Study Characteristics and Activities

Characteristic or activity	COMBO study	ESPI study and ESPI COMBO substudy
Study design	Longitudinal prospective cohort study; cross-sectional study	Prospective cohort study
Source population	Columbia University Irving Medical Center, New York, New York	Columbia University Irving Medical Center, New York, New York; University of Alabama, Birmingham; University of Utah, Salt Lake City
Range of infant birth dates	April 2020 to May 2021	January to September 2021
Enrollment dates	May 26, 2020, through present (ongoing)	ESPI: May 7 to November 3, 2021
		ESPI COMBO: August 2020 to March 2021
Eligibility criteria	Mothers received prenatal care and delivered at the Columbia University Irving Medical Center, New York–Presbyterian Morgan Stanley Children’s Hospital, or New York Presbyterian Allen Pavilion Hospital; maternal age ≥18 y; infant gestational age ≥18 wk	ESPI: gestation <28 wk; maternal age 18-50 y; willing to self-collect and mail swab specimens and respond to weekly surveillance contacts; willing to have data collected from infant’s health records; able to speak and read in English or Spanish
		ESPI COMBO: adherent to study surveillance activities for >40% of their enrollment wk; infants were aged <6 mo during the enrollment period
Ascertainment and testing	For all delivering patients: nasopharyngeal specimen PCR (March 22, 2020, and after) and serological testing for SARS-CoV-2 antibodies (July 20, 2020, and after)	Responded to weekly text messages about symptoms of COVID-19–like illness, received weekly midturbinate nasal swabs for SARS-CoV-2 PCR testing, and provided up to 3 serum samples for SARS-CoV-2 antibody testing (at enrollment, end of second trimester, and end of pregnancy)
Methods for determining prenatal SARS-CoV-2 exposure	Review of automated abstraction of data from EHRs; manual review of EHRs	Review of self-reported diagnoses of maternal infection before enrollment; PCR and serological testing as part of study procedures
Criteria for classification of exposure to maternal SARS-CoV-2 infection during pregnancy	Birth before November 1, 2020: PCR or serological positivity during pregnancy documented in EHR	Maternal PCR positivity documented by maternal self-report either before or after enrollment but during pregnancy plus serological evidence at study enrollment or evidence of maternal seroconversion from study enrollment through end of pregnancy
	Birth after November 1, 2020: PCR or antigen positivity during pregnancy	
	Mother with PCR positivity at birth: infant considered exposed during third trimester	
Criteria for classification of exposure to maternal SARS-CoV-2 infection before pregnancy or at an indeterminate time	NA	Maternal self-report of infection before pregnancy and maternal serological positivity at enrollment or serological positivity at entry into ESPI if exact timing of infection could not be determined
Criteria for classification of no exposure to maternal SARS-CoV-2 infection before or during pregnancy	Births before July 20, 2020: absence of PCR positivity in the EHR, absence of self-reported PCR positivity, absence of COVID-19 symptoms documented in EHR, absence of self-reported COVID-19 symptoms (information about potential for misclassification is available in eMethods 2 in Supplement 1)	All maternal PCR and serological results were negative and negative serological result at delivery
	Births after July 20, 2020: absence of PCR positivity and negative serological result at delivery	

Abbreviations: COMBO, COVID-19 Mother Baby Outcomes; EHR, electronic health record; ESPI, Epidemiology of Severe Acute Respiratory Syndrome Coronavirus 2 in Pregnancy and Infancy; ESPI COMBO, Epidemiology of Severe Acute Respiratory Syndrome Coronavirus 2 in Pregnancy and Infancy plus COMBO substudy; NA, not applicable; PCR, polymerase chain reaction.

### Determination of SARS-CoV-2 Exposure

Methods for determining SARS-CoV-2 exposure of infants enrolled in the COMBO study and the potential for misclassification are available in Table 1 and eMethods 2 in Supplement 1. CUIMC implemented clinical universal nasopharyngeal polymerase chain reaction (PCR) testing for all delivering patients on March 22, 2020, and universal serological testing for SARS-CoV-2 antibodies for all delivering patients on July 20, 2020.^16,24^ Infants born before November 1, 2020, were considered exposed during pregnancy if the mother had a positive SARS-CoV-2 PCR and/or serological test during pregnancy or at delivery, identified through automated abstraction of data from the electronic health record (EHR) system. Manual EHR review was performed to assess symptom status (asymptomatic vs symptomatic) and date of onset, from which trimester of exposure was determined. Mothers with a positive SARS-CoV-2 PCR test at delivery were considered exposed during the third trimester of pregnancy. Beginning on November 1, 2020, a positive serological test was insufficient to determine whether the infection occurred during or before the pregnancy. Therefore, infants born after this time were considered exposed only if the mother had a positive PCR or antigen test during pregnancy. Infants were considered unexposed if all PCR and serological tests available in the EHR for the mother were negative, which was estimated to have a 0.67% false-negative rate (eMethods 2 in Supplement 1).

The SARS-CoV-2 exposure status of infants in the ESPI COMBO cohort was determined through a combination of maternal self-report of COVID-19 diagnosis before enrollment and molecular and serological testing through the ESPI study. Infants were classified as exposed if their mother had SARS-CoV-2 infection detected from study surveillance samples or had evidence of seroconversion from study enrollment through the end of the pregnancy. Detailed methods for classification of exposure status, including the definition of seroconversion, are provided in eMethods 3 in Supplement 1. Exposure timing and maternal symptom status were determined through weekly text message surveillance for COVID-19–like illness symptoms, as previously described.^9^ Infants were considered unexposed if all maternal PCR and serological tests were negative.

A second exposed group, comprising only ESPI COMBO participants for whom the timing of SARS-CoV-2 exposure occurred before pregnancy or was indeterminate, was included in our analyses. Mothers in this group either had (1) a confirmed SARS-CoV-2 infection before pregnancy based on self-report and serological positivity at enrollment in the ESPI study or (2) a SARS-CoV-2 infection before conception or in early pregnancy based on serological positivity at enrollment in the ESPI study but with an indeterminate time of infection.

The exact timing of maternal SARS-CoV-2 infection could be determined for 91 of 112 infants (81.3%) exposed during pregnancy. Of those, 14 infants (15.4%) were exposed in the first trimester, 41 (45.1%) were exposed in the second trimester, and 36 (39.6%) were exposed in the third trimester. Maternal symptom status (asymptomatic vs symptomatic) was determined for 106 of 112 infants (94.6%) exposed during pregnancy. Of those, 27 infants (25.5%) were exposed to an asymptomatic maternal infection and 79 (74.5%) were exposed to a symptomatic maternal infection. All symptomatic infections were mild, with no mothers requiring hospitalization or oxygen supplementation.

### Infant Neurodevelopmental Assessment at Ages 5 to 11 Months

Infant neurodevelopment was assessed using the Developmental Assessment of Young Children, second edition (DAYC-2), between the corrected ages of 5 months, 15 days, and 11 months, 30 days (mean [SD] age, 8.0 [1.8] months). A total of 433 dyads from the COMBO study and 463 dyads from the ESPI COMBO substudy were invited to participate; of those, 152 dyads (35.1%) from the COMBO study and 299 dyads (64.6%) from the ESPI COMBO substudy completed the assessment between March 2021 and June 2022. Among the 299 dyads from the ESPI COMBO substudy, 281 dyads (65 from CUIMC, 40 from the University of Alabama, and 176 from the University of Utah) had available score forms. The DAYC-2 is a standardized assessment used in clinical and research settings. It has been normed using a national sample of 1832 children, has a population mean (SD) score of 100 (15), and provides age-adjusted standard scores for each subdomain. The DAYC-2 is typically conducted in person, including in the child’s home environment. Due to pandemic-related social distancing stipulations, research assistants (RAs) who were blinded to the dyad’s exposure status conducted the DAYC-2 in English or Spanish via telehealth visits (using the Zoom web-based video and meeting platform; Zoom Video Communications, Inc) while participants were at home (eFigure in Supplement 1). Research kits with standardized objects were mailed to participants for the assessment, which took approximately 45 minutes to complete. To reduce the potential for distraction, RAs turned off their cameras, and participants were directed to dim their screens. Items on the DAYC-2 can be scored using 3 approaches: direct observation of the child’s behavior, interview of the caregiver, and direct assessment. For items requiring prompts, the RA instructed the mother to prompt specific behaviors (eFigure in Supplement 1). The same RAs conducted the assessments across all 3 study sites.

A total of 26 infants were excluded from the analyses; 4 of these excluded infants were assessed for training purposes, 18 were assessed outside of the predefined age range, 3 were assessed by an RA who administered fewer than 1% of the assessments, and 1 had a poor testing environment (Figure 1). Some infants included in this analysis did not complete all of the DAYC-2 subdomains (eTable 1 in Supplement 1). Raw scores were converted into standard scores using the PRO-ED DAYC-2 Online Scoring and Report System (PRO-ED, Inc).

### Statistical Analysis

Statistical analyses were conducted using R software, version 4.1.3 (R Foundation for Statistical Computing), with deidentified data. Sociodemographic characteristics of each exposed group were compared with those of the unexposed group using logistic regression models for categorical variables and linear regression models for continuous variables, for which we reported mean differences (MDs) or odds ratios (ORs) with 95% CIs. Initial analyses consisted of linear regression models to estimate the main effects of exposure for each of 5 DAYC-2 subdomain scores (cognitive, gross motor, fine motor, expressive language, and receptive language). For each subdomain, we conducted linear regression models with the unexposed group as the reference group; each of the 2 exposed groups was separately compared with the unexposed group. For infants exposed during pregnancy, we examined the associations of trimester and symptom status of maternal infection with DAYC-2 scores by comparing each with the unexposed group.

We implemented unadjusted and adjusted models in which we controlled for variables that are suspected to be associated with infant neurodevelopment or that may have been confounders, including site (CUIMC, University of Alabama, or University of Utah), maternal self-reported race (Asian or Asian American [hereafter, Asian], Black or African American [hereafter, Black], Native American or Alaska Native, Native Hawaiian or other Pacific Islander, White, or other or multiple races) and ethnicity (Hispanic, Latinx, or Spanish [hereafter, Hispanic] or non-Hispanic, non-Latinx, or non-Spanish [hereafter, non-Hispanic), age at delivery, insurance status (commercial or Medicaid), parity (multiparous or primiparous), mode of delivery (vaginal or cesarean), infant gestational age at birth, infant sex assigned at birth (female or male), and assessment language (English or Spanish). Race and ethnicity categories are reported in this article given known COVID-19 racial and ethnic disparities; however, race and ethnicity were not used for inclusion criteria or as a selection strategy. All participating mothers self-reported their race and ethnicity through study surveys. Missing covariate data are reported in Table 2. Missing categorical data were handled using the missing indicator method.^25^ There were no missing data for continuous variables. For all models, we reported standardized regression coefficients (β values) and SEs of estimates for each main effect in adjusted models. The significance level was set at *P* = .05. A power analysis for the primary outcome was conducted (eMethods 4 in Supplement 1).

**Table 2.** Demographic and Clinical Characteristics of Study Sample

Characteristic	Participants, No. (%)				Exposed during pregnancy vs unexposed		Exposed before pregnancy or at indeterminate time vs unexposed	
	Total	Unexposed	Exposed during pregnancy	Exposed before pregnancy or at indeterminate time				
					MD or OR (95% CI)	*P* value	β or OR (95% CI)	*P* value
**Mother**								
Total participants, No.	403	256	111	36	NA	NA	NA	NA
Age at delivery, y								
Mean (SD)	32.1 (5.4)	32.4 (5.4)	32.0 (5.4)	30.5 (5.3)	−0.44 (−1.64 to 0.76)	.48	−1.92 (−1.31 to −0.07)^a^	.04
Median (range)	32.0 (18.7 to 46.0)	32.4 (19.0 to 45.0)	31.0 (22.0 to 46.0)	31.3 (18.7 to 39.2)				
Primiparous	182 (45.2)	124 (48.4)	44 (39.6)	14 (38.9)	0.70 (0.44 to 1.10)^b^	.12	0.68 (0.33 to 1.37)^c^	.29
Self-reported race								
Asian or Asian American	13 (3.2)	12 (4.7)	1 (0.9)	0	0.15 (0.01 to 0.96)^b^	.12	NA^d^	>.99
Black or African American	45 (11.2)	23 (9.0)	15 (13.5)	7 (19.4)	1.87 (0.96 to 3.60)^b^	.06	2.33 (0.87 to 5.67)^c^	.07
Native American or Alaska Native	6 (1.5)	3 (1.2)	3 (2.7)	0	2.36 (0.55 to 10.13)^b^	.23	NA^d^	>.99
Native Hawaiian or other Pacific Islander	3 (0.7)	0	3 (2.7)	0	7.08 (0.90 to 144.07)^b^	.09	NA^d^	>.99
White	240 (59.6)	169 (66.0)	50 (45.0)	21 (58.3)	0.41 (0.26 to 0.65)^b^	<.001	0.71 (0.35 to 1.47)^c^	.34
Other or multiple races	45 (11.2)	22 (8.6)	19 (17.1)	4 (11.1)	2.23 (1.16 to 4.25)^b^	.02	1.27 (0.35 to 3.56)^c^	.68
Declined to answer or unknown	51 (12.7)	27 (10.5)	20 (18.0)	4 (11.1)	2.13 (0.78 to 5.73)^b^	.13	3.43 (0.89 to 11.21)^c^	.05
Self-reported ethnicity								
Hispanic, Latinx, or Spanish	144 (35.7)	66 (25.8)	56 (50.5)	22 (61.1)	2.93 (1.84 to 4.68)^b^	<.001	4.52 (2.21 to 9.55)^c^	<.001
Not Hispanic, Latinx, or Spanish	253 (62.8)	185 (72.3)	54 (48.6)	14 (38.9)	0.36 (0.23 to 0.58)^b^	<.001	0.24 (0.12 to 0.50)^c^	<.001
Declined to answer or unknown	6 (1.5)	5 (2.0)	1 (0.9)	0	0.46 (0.02 to 2.87)^b^	.48	NA^d^	NA
Insurance status								
Commercial	263 (65.3)	187 (73.0)	60 (54.1)	16 (44.4)	0.43 (0.27 to 0.69)^b^	<.001	0.30 (0.14 to 0.60)^c^	<.001
Medicaid	136 (33.7)	66 (25.8)	50 (45.0)	20 (55.6)	2.36 (1.48 to 3.77)^b^	<.001	3.60 (1.77 to 7.45)^c^	<.001
Unknown	4 (1.0)	3 (1.2)	1 (0.9)	0	0.77 (0.04 to 6.06)^b^	.82	NA^d^	>.99
**Infant**								
Total participants, No.	407	258	112	37	NA	NA	NA	NA
GA at birth, wk								
Mean (SD)	38.7 (1.7)	38.8 (1.5)	38.5 (2.1)	38.3 (2.0)	−0.28 (−0.66 to 0.10)^a^	.14	−0.56 (−1.11 to −0.01)^c^	.05
Median (range)	39.1 (28.9 to 41.6)	39.1 (29.6 to 41.6)	39.1 (28.9 to 41.1)	39.0 (32.0 to 41.0)				
Preterm birth (GA <37 wk)	40 (9.8)	19 (7.4)	17 (15.2)	4 (10.8)	0.44 (0.22 to 0.90)^b^	.02	0.66 (0.23 to 2.37)^c^	.47
Sex								
Female	195 (47.9)	122 (47.3)	55 (49.1)	18 (48.6)	1.08 (0.69 to 1.68)^b^	.75	1.06 (0.53 to 2.11)^c^	.88
Male	212 (52.1)	136 (52.7)	57 (50.9)	19 (51.4)				
Vaginal delivery	266 (65.4)	164 (63.6)	76 (67.9)	26 (70.3)	1.21 (0.76 to 1.95)^b^	.43	1.35 (0.66 to 2.97)^c^	.43
Twin	9 (2.2)	4 (1.6)	3 (2.7)	2 (5.4)	1.75 (0.34 to 8.05)^b^	.47	3.63 (0.49 to 19.31)^c^	.15
Age at assessment, mo								
Mean (SD)	8.0 (1.8)	7.9 (1.8)	8.5 (1.8)	7.2 (1.5)	0.60 (0.20 to 1.00)^b^	.003	−0.75 (−1.36 to −0.14)^c^	.02
Median (range)	8.0 (5.0 to 11.0)	8.0 (5.0 to 11.0)	9.0 (5.0 to 11.0)	7.0 (5.0 to 11.0)				
Assessed in Spanish	66 (16.2)	27 (10.5)	26 (23.2)	13 (35.1)	2.59 (1.43 to 4.69)^b^	.002	4.63 (2.08 to 10.08)^c^	<.001

Abbreviations: GA, gestational age; MD, mean difference; NA, not applicable; OR, odds ratio.

^a^ MD values (reported for continuous data).

^b^ OR values (reported for categorical data).

^c^ β Values.

^d^ NA applies to OR values in which 1 side of the comparison included 0 participants.

## Results

### Cohort Characteristics

The sample consisted of 407 infants (258 [63.4%] unexposed, 112 [27.5%] exposed during pregnancy, and 37 [9.1%] exposed before pregnancy or at an indeterminate time) born to 403 mothers (256 [63.5%] unexposed, 111 [27.5%] exposed during pregnancy, and 36 [8.9%] exposed before pregnancy or at an indeterminate time) (Table 2). The mean (SD) maternal age at delivery was 32.1 (5.4) years. With regard to race, 13 mothers (3.2%) were Asian, 45 (11.2%) were Black, 6 (1.5%) were Native American or Alaska Native, 3 (0.7%) were Native Hawaiian or other Pacific Islander, 240 (59.6%) were White, 45 (11.2%) were of other or multiple races, and 51 (12.7%) declined to answer or were of unknown race. With regard to ethnicity, 144 mothers (35.7%) were Hispanic, 253 (62.8%) were non-Hispanic, and 6 (1.5%) declined to answer or were of unknown ethnicity. The majority of infants (367 [90.2%]) were born full term; 195 infants (47.9%) were female and 212 (52.1%) were male.

Several sociodemographic characteristics were compared between the 2 exposed groups and the unexposed group. The group exposed during pregnancy had a lower proportion of White mothers (OR, 0.41; 95% CI, 0.26-0.65) and a higher proportion of mothers who were of other or multiple races (OR, 2.23; 95% CI, 1.16-4.25) or Hispanic ethnicity (OR, 2.93; 95% CI, 1.84-4.68) compared with the unexposed group. The group with prepregnancy or indeterminate time of prenatal exposure had a higher proportion of Hispanic mothers (OR, 4.52; 95% CI, 2.21-9.55) than the unexposed group, and 56 of 144 Hispanic mothers (38.9%) and 54 of 253 non-Hispanic mothers (21.3%) had SARS-CoV-2 infection during pregnancy. The prevalence of infection was 15.3% (22 of 144) for Hispanic mothers and 5.5% (14 of 253) for non-Hispanic mothers with prepregnancy or indeterminate time of exposure. Mothers with commercial insurance were less likely to have an infection during pregnancy (OR, 0.43; 95% CI, 0.27-0.69) or before pregnancy or at an indeterminate time (OR, 0.30; 95% CI, 0.14-0.60). Unexposed infants were less likely to be born preterm than those exposed before pregnancy or at an indeterminate time (OR, 0.66; 95% CI, 0.23-2.37), and infants exposed before pregnancy or at an indeterminate time had lower gestational ages (MD, −1.92; 95% CI, −1.31 to −0.07) than unexposed infants. Infants exposed during pregnancy were slightly older than unexposed infants at the assessment (MD, 0.60; 95% CI, 0.20-1.00), and those with mothers who had an infection before pregnancy or at an indeterminate time were slightly younger (MD, −0.75; 95% CI, −1.36 to −0.14) than unexposed infants. A higher proportion of infants who were exposed during pregnancy (OR, 2.59; 95% CI, 1.43-4.69) or exposed before pregnancy or at an indeterminate time (OR, 4.63; 95% CI, 2.08-10.08) were assessed in Spanish compared with infants who were unexposed. Maternal and infant demographic characteristics and DAYC-2 scores differed significantly by site (eResults, eTable 2, and eTable 3 in Supplement 1), which was accounted for in fully adjusted models. For example, unadjusted pairwise comparisons revealed that DAYC-2 cognitive (β = 4.44; 95% CI, 0.93-7.96), fine motor (β = 1.58; 95% CI, 0.45-2.72), and expressive language (β = 3.77; 95% CI, 0.72-6.83) subdomain scores were higher among infants at the University of Utah compared with those at CUIMC.

### Association of Maternal SARS-CoV-2 Infection With Differences in DAYC-2 Scores

**Figure 2.**
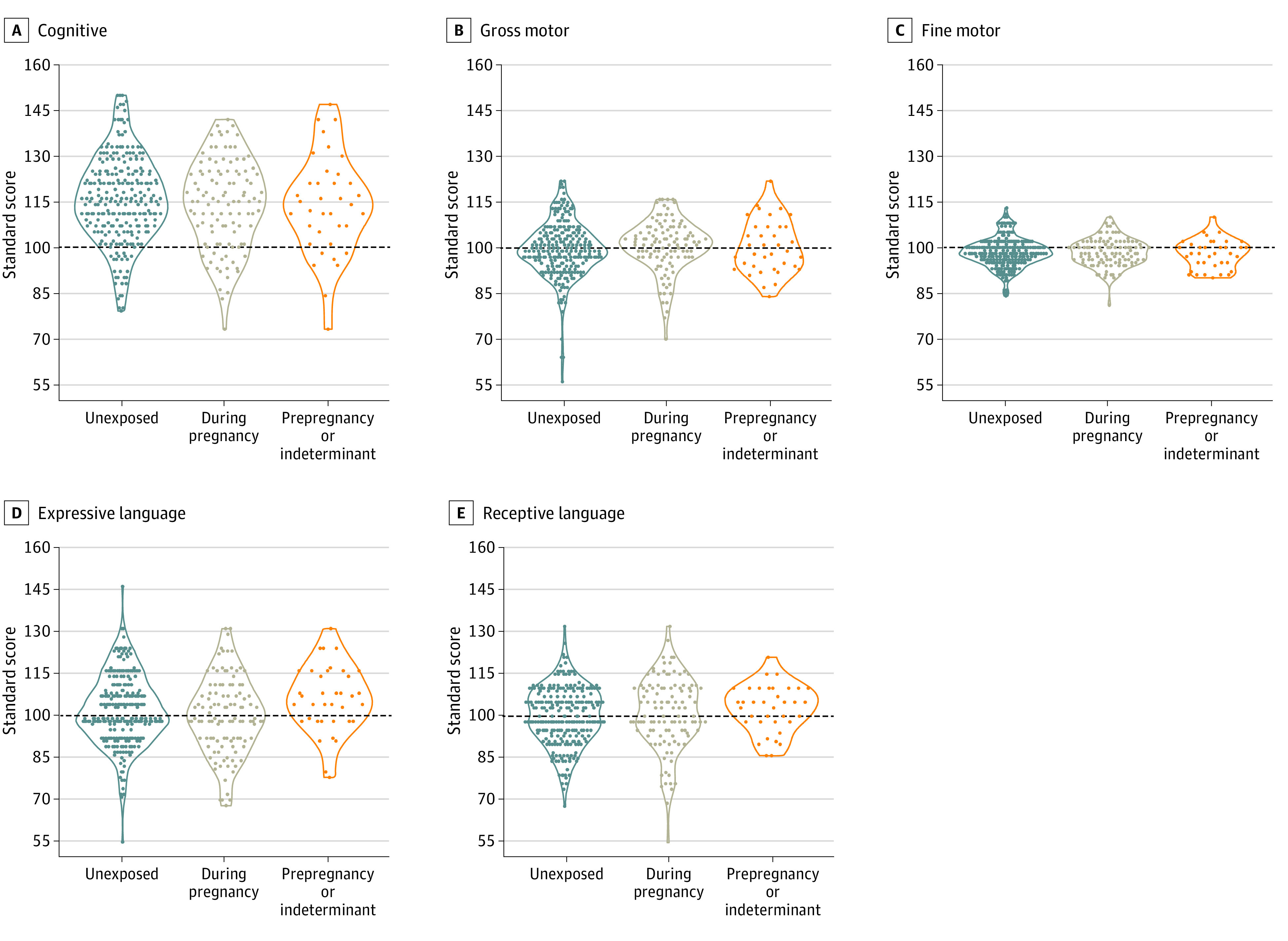
Unadjusted Standard Scores for Each DAYC-2 Subdomain Horizontal dashed lines represent the normative mean score for each subdomain. DAYC-2 indicates Developmental Assessment of Young Children, second edition.

There was no association between exposure to maternal SARS-CoV-2 infection during pregnancy and DAYC-2 cognitive, gross motor, fine motor, expressive language, or receptive language subdomain scores based on comparison with unexposed infants in either unadjusted models (cognitive: β = −1.49 [95% CI, −4.70 to 1.71; *P* = .36]; gross motor: β = 1.09 [95% CI, −0.95 to 3.12; *P* = .29]; fine motor: β = −0.01 [95% CI, −1.06 to 1.04; *P* = .98]; expressive language: β = −1.96 [95% CI, −4.78 to 0.87; *P* = .17]; and receptive language: β = 0.87 [95% CI, −1.60 to 3.35; *P* = .49]) (Table 3 and Figure 2) or adjusted models (cognitive: β = 0.31 [95% CI, −2.97 to 3.58; *P* = .85]; gross motor: β = 0.82 [95% CI, −1.34 to 2.99; *P* = .46]; fine motor: β = 0.36 [95% CI, −0.74 to 1.47; *P* = .52]; expressive language: β = −1.00 [95% CI, −4.02 to 2.02; *P* = .51]; and receptive language: β = 0.45 [95% CI, −2.15 to 3.04; *P* = .74]) (Table 3). Similarly, there was no association between exposure to maternal SARS-CoV-2 infection before pregnancy or at an indeterminate time and DAYC-2 cognitive, gross motor, fine motor, expressive language, or receptive language subdomain scores compared with unexposed infants in unadjusted models (cognitive: β = −1.86 [95% CI, −6.84 to 3.12; *P* = .46]; gross motor: β = 0.89 [95% CI, −2.27 to 4.04; *P* = .58]; fine motor: β = −0.52 [95% CI, −2.16 to 1.12; *P* = .53]; expressive language: β = 4.11 [95% CI, −0.10 to 8.30; *P* = .06]; and receptive language: β = 2.66 [95% CI, −0.84 to 6.16; *P* = .14]) (Table 3 and Figure 2) or adjusted models (cognitive: β = 1.65 [95% CI, −3.63 to 6.92; *P* = .54]; gross motor: β = 0.75 [95% CI, −2.62 to 4.12; *P* = .66]; fine motor: β = −0.25 [95% CI, −1.98 to 1.49; *P* = .78]; expressive language: β = 3.83 [95% CI, −0.71 to 8.38; *P* = .10]; and receptive language: β = 2.96 [95% CI, −0.75 to 6.66; *P* = .12]) (Table 3).

**Table 3.** Comparison of DAYC-2 Subdomain Scores by Timing of Exposure to SARS-CoV-2 Infection

DAYC-2 subdomain	DAYC-2 score, mean (SD)				Exposed during pregnancy vs unexposed				Exposed before pregnancy or at indeterminate time vs unexposed			
	All participants (N = 407)	Unexposed (n = 258)	Exposed during pregnancy (n = 112)	Exposed before pregnancy or at indeterminate time (n = 37)	Unadjusted		Adjusted^a^		Unadjusted		Adjusted^a^	
					β (95% CI)	*P* value	β (95% CI)	*P* value	β (95% CI)	*P* value	β (95% CI)	*P* value
Cognitive	115.1 (14.4)	115.7 (14.1)	114.2 (14.5)	113.8 (16.2)	−1.49 (−4.70 to 1.71)	.36	0.31 (−2.97 to 3.58)	.85	−1.86 (−6.84 to 3.12)	.46	1.65 (−3.63 to 6.92)	.54
Gross motor	100.0 (9.0)	99.6 (9.2)	100.7 (8.6)	100.5 (8.7)	1.09 (−0.95 to 3.12)	.29	0.82 (−1.34 to 2.99)	.46	0.89 (−2.27 to 4.04)	.58	0.75 (−2.62 to 4.12)	.66
Fine motor	98.3 (4.7)	98.4 (4.7)	98.4 (4.5)	97.9 (4.8)	−0.01 (−1.06 to 1.04)	.98	0.36 (−0.74 to 1.47)	.52	−0.52 (−2.16 to 1.12)	.53	−0.25 (−1.98 to 1.49)	.78
Expressive language	101.8 (12.6)	102.0 (12.2)	100.0 (13.6)	106.1 (11.7)	−1.96 (−4.78 to 0.87)	.17	−1.00 (−4.02 to 2.02)	.51	4.11 (−0.10 to 8.30)	.06	3.83 (−0.71 to 8.38)	.10
Receptive language	100.6 (10.9)	100.1 (10.2)	101.0 (12.9)	102.8 (8.4)	0.87 (−1.60 to 3.35)	.49	0.45 (−2.15 to 3.04)	.74	2.66 (−0.84 to 6.16)	.14	2.96 (−0.75 to 6.66)	.12

Abbreviation: DAYC-2, Developmental Assessment of Young Children, second edition.

^a^ Adjusted model included maternal race, maternal ethnicity, maternal age at birth, insurance status, parity, delivery method, infant’s gestational age at birth, infant’s sex, study site, and language of assessment.

### Association of Trimester of Maternal SARS-CoV-2 Infection and Symptom Status During Pregnancy With Differences in DAYC-2 Scores

The trimester of maternal SARS-CoV-2 infection was not associated with DAYC-2 subdomain scores. Compared with nonexposure, infant exposure to maternal SARS-CoV-2 infection during the first, second, or third trimester of pregnancy was not associated with cognitive, gross motor, fine motor, expressive language, or receptive language scores in either unadjusted or adjusted models (eTable 4 in Supplement 1).

Similarly, maternal symptom status during pregnancy was not associated with DAYC-2 cognitive, fine motor, expressive language, or receptive language scores in unadjusted or adjusted models (eTable 5 in Supplement 1). Of note, exposure to asymptomatic infection was associated with higher DAYC-2 gross motor scores compared with nonexposure in the unadjusted model (β = 4.54; 95% CI, 0.90-8.19; *P* = .01), and this finding remained significant in the adjusted model (β = 4.42; 95% CI, 0.61-8.22; *P* = .02) (eTable 5 in Supplement 1). There was no association between exposure to symptomatic infection and DAYC-2 gross motor scores in the unadjusted or adjusted models (eTable 5 in Supplement 1).

## Discussion

To our knowledge, this cohort study implemented the first standardized telehealth-adapted observer-based neurodevelopmental assessment of infants born during the COVID-19 pandemic with and without prenatal exposure to asymptomatic or mild maternal SARS-CoV-2 infection across 3 distinct geographic regions within the US. Previous reports^4,16,26^ of the association between prenatal exposure to SARS-CoV-2 infection and neurodevelopment have generated conflicting findings. Two previous studies,^16,26^ including one from the COMBO Initiative,^16^ found no association between SARS-CoV-2 exposure during pregnancy and neurodevelopment within the first 12 months using parental report measures. However, a 2020 analysis^4^ of *International Statistical Classification of Diseases and Related Health Problems, Tenth Revision*, diagnostic codes abstracted from medical records suggested that prenatally exposed infants were more likely to receive a neurodevelopmental diagnosis by age 12 months. Similar to previous COMBO Initiative findings using a parental report measure,^16^ the current analysis found that maternal SARS-CoV-2 infection during pregnancy was not associated with decrements in infant neurodevelopment. Neither the trimester of maternal infection during pregnancy nor maternal symptom status was associated with neurodevelopmental scores, with the exception of slightly higher gross motor scores in infants born to mothers with asymptomatic disease compared with infants not exposed to SARS-CoV-2 infection. Given the continued high prevalence of COVID-19, these findings offer information that may be helpful for pregnant individuals who might contract SARS-CoV-2 during pregnancy, although additional studies with longer-term follow-up of prenatally exposed infants are still needed.

This study addressed several limitations of previous studies by the COMBO initiative^16^ and other research groups.^19,20^ Previous work by the COMBO Initiative^16^ was limited to infants born in New York City during the initial wave of the COVID-19 pandemic, while the current analysis included infants born across 3 distinct geographic regions in the US. The previous study^16^ also relied on a parental report measure and was therefore subject to potential parental biases. The present study was, to our knowledge, the first to use the standardized observer-based DAYC-2 assessment conducted by RAs blinded to exposure status. In addition, our analysis exclusively considered prenatal exposure to SARS-CoV-2 infection. While we cannot rule out potential postnatal factors (eg, maternal psychological distress^27^), findings from a previous study^28^ suggested that postnatal exposure to pandemic-related disruptions was not associated with infant neurodevelopment.

### Limitations

This study has several limitations. Similar to limitations previously described by the COMBO Initiative,^16^ it is possible that unmeasured confounding variables, such as psychosocial factors, may have impacted our results. The DAYC-2 assessment was administered between ages 5 months, 15 days, and 11 months, 30 days, which represents a wide range of early developmental time points. Further follow-up of these children is needed to evaluate the potential associations between prenatal SARS-CoV-2 exposure and longer-term clinically important outcomes. Our results cannot be generalized to infants exposed to moderate to severe maternal SARS-CoV-2 infections during pregnancy because the sample includes only infants with mothers who had asymptomatic or mild infections. While the primary unadjusted model was appropriately powered, some adjusted models and those used for subanalyses may be underpowered. In the COMBO cohort, there is potential for misclassification of exposure status because this was not a prospective surveillance cohort. In addition, the length of DAYC-2 assessments, which required approximately 45 minutes to complete, is a limitation.

We conducted the DAYC-2 via a web-based video platform (Zoom), which deviates from standard administration, and the lack of validation of this mode of administration is a substantial limitation. However, this modification confers several important benefits,^29^ including the observation of infants in a naturalistic home environment, which may represent the infant’s behavior more accurately than what is typically observed in a clinic. Had we conducted the assessment in person, research personnel would have been required to use personal protective equipment, including masks and face shields, which may have had substantial implications for performance on the assessment. In addition, the DAYC-2 can be administered remotely for clinical purposes,^30^ and the CUIMC neonatal follow-up clinic began administering the DAYC-2 via telehealth visits at the beginning of the pandemic.

## Conclusions

This cohort study used a standardized observational measure and provided the first evidence to date that prenatal exposure to SARS-CoV-2 infection is not associated with differences in neurodevelopment between ages 5 and 11 months. The results did not reveal an association between the timing or symptom status of asymptomatic and mild maternal SARS-CoV-2 infection during pregnancy and infant neurodevelopmental scores.

## Notes

Supplement 1. **eMethods 1.** Study Cohort Descriptions **eMethods 2.** SARS-CoV-2 Classification and Potential for Misclassification in the COMBO Study **eMethods 3.** Classification of SARS-CoV-2 Status in the ESPI COMBO Substudy **eMethods 4.** Power Analysis **eResults.** Association of Enrollment Site With DAYC-2 Scores at Ages 6 to 11 Months **eFigure.** Modified DAYC-2 Assessment Conducted Through a Video Visit **eTable 1.** Cause of Missing Scores in Each DAYC-2 Subdomain **eTable 2.** Demographic and Clinical Characteristics of Study Sample by Site **eTable 3.** Comparison of DAYC-2 Subdomain Scores Across Enrollment Sites **eTable 4.** Comparison of DAYC-2 Subdomain Scores Between SARS-CoV-2 Nonexposure, First Trimester Exposure, Second Trimester Exposure, and Third Trimester Exposure **eTable 5.** Comparison of DAYC-2 Subdomain Scores Between SARS-CoV-2 Negativity During Pregnancy, Asymptomatic Infection During Pregnancy, and Symptomatic Infection During Pregnancy
eReferences

Supplement 2.
Data Sharing Statement
