# Supplementary material for: Assessment of Neurodevelopment in Infants With and Without Exposure to Asymptomatic or Mild Maternal SARS-CoV-2 Infection During Pregnancy
Source: JAMA Netw Open. 2023 Apr 10;6(4):e237396. doi: 10.1001/jamanetworkopen.2023.7396 (PMC10087058; doi:10.1001/jamanetworkopen.2023.7396)
Supplement: Supplement 1. — eMethods 1. Study Cohort Descriptions eMethods 2. SARS-CoV-2 Classification and Potential for Misclassification in the COMBO Study eMethods 3. Classification of SARS-CoV-2 Status in the ESPI COMBO Substudy eMethods 4. Power Analysis eResults. Association of Enrollment Site With DAYC-2 Scores at Ages 6 to 11 Months eFigure. Modified DAYC-2 Assessment Conducted Through a Video Visit eTable 1. Cause of Missing Scores in Each DAYC-2 Subdomain eTable 2. Demographic and Clinical Characteristics of Study Sample by Site eTable 3. Comparison of DAYC-2 Subdomain Scores Across Enrollment Sites eTable 4. Comparison of DAYC-2 Subdomain Scores Between SARS-CoV-2 Nonexposure, First Trimester Exposure, Second Trimester Exposure, and Third Trimester Exposure eTable 5. Comparison of DAYC-2 Subdomain Scores Between SARS-CoV-2 Negativity During Pregnancy, Asymptomatic Infection During Pregnancy, and Symptomatic Infection During Pregnancy eReferences [file jamanetwopen-e237396-s001.pdf]

Supplementary Online Content

Firestein MR, Shuffrey LC, Hu Y, et al. Assessment of neurodevelopment in infants with and without exposure to asymptomatic or mild maternal SARS-CoV-2 infection during pregnancy. *JAMA Netw Open*. 2023;6(4):e237396. doi:10.1001/jamanetworkopen.2023.7396

eMethods 1. Study Cohort Descriptions

eMethods 2. SARS-CoV-2 Classification and Potential for Misclassification in the COMBO Study

eMethods 3. Classification of SARS-CoV-2 Status in the ESPI COMBO Substudy

eMethods 4. Power Analysis

eResults. Association of Enrollment Site With DAYC-2 Scores at Ages 6 to 11 Months

eFigure. Modified DAYC-2 Assessment Conducted Through a Video Visit

eTable 1. Cause of Missing Scores in Each DAYC-2 Subdomain

eTable 2. Demographic and Clinical Characteristics of Study Sample by Site

eTable 3. Comparison of DAYC-2 Subdomain Scores Across Enrollment Sites

eTable 4. Comparison of DAYC-2 Subdomain Scores Between SARS-CoV-2 Nonexposure, First Trimester Exposure, Second Trimester Exposure, and Third Trimester Exposure

eTable 5. Comparison of DAYC-2 Subdomain Scores Between SARS-CoV-2 Negativity During Pregnancy, Asymptomatic Infection During Pregnancy, and Symptomatic Infection During Pregnancy[1]

eReferences

This supplemental material has been provided by the authors to give readers additional information about their work.

© 2023 Firestein MR et al. *JAMA Network Open*.

eMethods 1. Study Cohort Descriptions

COMBO Initiative Cohort

Patients who receive prenatal care and deliver at the Columbia University Irving Medical Center (CUIMC)-affiliated NewYork-Presbyterian (NYP) Morgan Stanley Children's Hospital (MSCH) or NYP Allen Pavilion Hospital are eligible to participate in COMBO. The recruitment and enrollment strategy for the COMBO Initiative has previously been described[1] and remains ongoing. Since May 2020, pregnant women with a documented SARS-CoV-2 infection during pregnancy (exposed group) are invited to participate beginning prenatally or at any of the existing study timepoints. CUIMC implemented universal nasopharyngeal PCR testing and universal serological testing for SARS-CoV-2 antibodies for all delivering patients on March 22 and July 20, 2020, respectively[1,2]. Infants born prior to November 1, 2020 were classified as exposed during pregnancy if the mother had a positive SARS-CoV-2 PCR and/or serology test during pregnancy or at delivery, identified through automated abstraction of data from the electronic health record (EHR) system, followed by manual EHR review to assess symptom status (asymptomatic vs. symptomatic) and date of onset, which was used to determine trimester of exposure. Infants whose mothers had a positive SARS-CoV-2 PCR test at delivery were considered exposed during the third trimester of pregnancy. After November 1, 2020, a positive serology test was insufficient to determine whether the infection occurred during or prior to the pregnancy and therefore, infants born after this time were considered to have been exposed if the mother had a positive PCR or antigen test during pregnancy. Infants were classified as 'unexposed' if all PCR and serology testing available in the EHR for the mother were negative, which was estimated to be associated with a 0.67% false negative rate (eMethods 2).

After delivery, each enrolled infant born to a mother with a documented infection during pregnancy is matched to 1-to-3 infants without documented exposure to a maternal SARS-CoV-2 infection during pregnancy (unexposed group). The unexposed group was defined as the absence of PCR positivity in the EHR, the absence of self-reported PCR positivity, the absence of COVID symptoms documented in EHR, and the absence of self-reported COVID symptoms for infants born prior to July 20,

© 2023 Firestein MR et al. *JAMA Network Open*.

2020, and the false negative rate associated with this classification was computed (eMethods 2). After July 20, 2020, the unexposed group was defined as the absence of PCR positivity and a negative serology test at the time of delivery. The exposed and unexposed groups are matched on infant sex, gestational age (GA) at birth, mode of delivery, and infant date of birth within a 2-week window. Maternal vaccination status during pregnancy was not systematically documented. The ongoing COMBO Initiative has enrolled 702 participants since May 2020, with primary data collection consisting of online surveys. Subsets of participants are invited to participate in various deep phenotyping assessments, including video visits described here. All study procedures were reviewed and approved by the CUIMC Institutional Review Board (IRB) and written informed consent for study procedures, including the use of video and photography footage, was obtained from all participants. Participants received financial compensation for all study procedures.

ESPI COMBO Cohort

Pregnant individuals less than 28 weeks' gestation who received care at three US academic medical centers (CUIMC, University of Alabama (UAB) and University of Utah (UU)) were invited to participate in the CDC's ESPI Network Community Cohort, which prospectively enrolled pregnant individuals between August 2020 and March 2021 to study the incidence of SARS-CoV-2 infections in this population. Participants self-reported whether they were diagnosed with COVID-19 by a healthcare professional before study enrollment, engaged in surveillance for SARS-CoV-2 infection which included responding to weekly text-messages about symptoms of COVID-19-like illness, submitting weekly mid-turbinate nasal swabs for SARS-CoV-2 PCR testing, and providing up to three serum samples for SARS-CoV-2 antibody-testing. Once COVID-19 vaccines became available in the United States, participants also provided information about whether they received the COVID-19 vaccine during pregnancy and self-reported vaccination status was verified with electronic medical records or local vaccine registries when possible. The recruitment, enrollment, and surveillance outcomes for the ESPI Community Network cohort have been separately described[3]. Of the 1,169 individuals who were fully enrolled in ESPI and

© 2023 Firestein MR et al. *JAMA Network Open*.

participated in SARS-CoV-2 surveillance, 689 were invited to participate in the ESPI COMBO sub-study because they were adherent to study surveillance activities for >40% of their enrollment weeks and their infants were <6 months of age during the ESPI COMBO enrollment period. The ESPI COMBO sub-study mirrored COMBO data collection from delivery to 6 months postpartum. ESPI COMBO infants who contributed to analyses presented here were born between January 2021 and September 2021.

© 2023 Firestein MR et al. *JAMA Network Open*.

eMethods 2. SARS-CoV-2 Classification and Potential for Misclassification in the COMBO Study

The COMBO Initiative utilizes NYP's Clinical Data Warehouse to automatically extract data, including maternal SARS-CoV-2 test results by PCR and serology, on all delivering mothers and their infants (once born) every two weeks. Following automated EHR extraction of mothers infected with SARS-CoV-2 infections during pregnancy, a detailed chart review was conducted for each exposed mother to determine symptom severity and timing. This chart review was primarily conducted by pediatricians and obstetricians providing patient care during the pandemic, or research assistants trained by these clinicians. For women identified as exposed by serology only, the chart was reviewed for outside records scanned into our EHR system, in person and telehealth visits, and all other notes between onset of the pandemic (early March 2020) and birth. The occurrence of a SARS-CoV-2 infection during pregnancy was determined by PCR test results with or without serology for 21 (32.8%), by serology for 42 (65.6%), and by self-report and EHR documentation of non-specific outside positive COVID testing for 1 (1.6%) of the exposed participants from the COMBO cohort included in this analysis. Given that false positive SARS-CoV-2 results by both PCR and serology are extremely rare and most of our patients had either multiple positive tests or experienced symptoms, the potential for misclassifying unexposed women into the exposed group is very low and was not considered in our exploration of effects of misclassification. Based on the detailed chart review, the SARS-CoV-2 infection was classified as either asymptomatic or symptomatic.

Chart review was also conducted for dyads selected for approach for enrollment into the unexposed group and if any evidence of COVID-19 was found during pregnancy, the mother was not selected or was reclassified to the exposed group. However, two sources of misclassification exist: asymptomatic disease and nonseroconversion. The asymptomatic disease in our population can be estimated as follows: 19 (30%) of 64 exposed women in our cohort were asymptomatic, a rate consistent with reports from larger cohorts of pregnant women[4]. The most conservative estimate of misclassification of exposed dyads into the unexposed group prior to 7/20/2020 (when universal testing by serology was

© 2023 Firestein MR et al. *JAMA Network Open*.

initiated) is therefore 30% of 15% of women expected to have COVID-19 disease at some point in pregnancy[5], which results in 4.5%. After 7/20/2020, misclassification would occur most likely due to asymptomatic disease in nonseroconverters. Therefore, the most conservative rate of misclassification would be 15% of the 4.5% of asymptomatic women or 0.67% of all women initially screened into the unexposed group.

© 2023 Firestein MR et al. *JAMA Network Open*.

eMethods 3. Classification of SARS-CoV-2 Status in the ESPI COMBO Substudy

Classification was based on a combination of maternal self-report of COVID positivity prior to ESPI enrollment, PCR and symptoms during the ESPI phase, and serological testing during the ESPI phase. The occurrence of a SARS-CoV-2 infection during pregnancy was determined by PCR test results for 36 (72%) and by serology for 14 (28%) of the exposed participants from the ESPI COMBO cohort included in this analysis.

Serological testing in ESPI was conducted from sera collected up to three times during pregnancy (at enrollment, at end of second trimester and at end of pregnancy). All participants had serological testing performed at the end of pregnancy. Samples were tested at the CDC using Luminex xMAP-SARS-CoV-2 Multi Antigen Assay, which is a qualitative assay. The assay tests against 3 antigens: S1, RBD, and nucleocapsid (N) proteins. The N protein is unique to natural infection, whereas S1 and RBD antibodies form in response to both natural infection and vaccination. Therefore, looking at all three antibodies helps differentiate between positive testing in response to vaccination versus natural infection. However, the ESPI sample contained individuals positive for S1 and/or RBD (negative for N), who had never been vaccinated, which was attributed to quicker waning of antibodies against N than of antibodies against S1 and RBD. Therefore, the following classification taking into account self-report, PCR results, and antibody positivity was implemented:

1. Control group = no *in utero* exposure
    - No self-reported COVID and
    - Absent positive PCR from ESPI enrollment to delivery and
    - Negative S1, RBD, N on all serological testing in ESPI and
    - Serology testing was obtained at the end of pregnancy and was negative

© 2023 Firestein MR et al. *JAMA Network Open*.

2. Pre-pregnancy = no *in utero* exposure
    - Self-reported COVID infection with a specific date prior to calculated conception date and
    - Serology consistent with prior infection:
        - Positive N at entry into ESPI or
        - Positive RBD/S1 at entry into ESPI and no history of vaccination
3. Unknown = possible *in utero* exposure
    - No self-report of COVID history, but:
        - Positive N at entry into ESPI or
        - Positive RBD/S1 and no history of vaccination
4. COVID pos = definite *in utero* exposure
    - PCR+ from ESPI enrollment to delivery or
    - Serological conversion from ESPI enrollment to delivery that cannot be explained by vaccination:
        - Serological conversion with N pos irrespective of vaccine and sx or
        - Serological conversion with RBD/S1 prior to vaccine irrespective of sx

© 2023 Firestein MR et al. *JAMA Network Open*.

eMethods 4. Power Analysis

Our power analyses were based on our primary outcome, which aimed to investigate differences in DAYC-2 subdomain scores between infants with and without *in utero* exposure to maternal SARS-CoV-2 infections. The DAYC-2 is a normative standardized assessment with a population mean score of 100 and standard deviation (SD) of 15. Using these normative values, our power analysis aimed to ensure 90% power to detect a 0.5 SD difference between groups, with alpha set to <0.05. Given the enrollment ratios of approximately 1:1 (COMBO) and 1:3 (ESPI COMBO), achieving this requires a sample size of a minimum of 168 or 225 infants, respectively, for all subdomains. The current manuscript includes a total of 403 infants and therefore our analyses were well-powered to detect differences in DAYC-2 subdomain scores of at least 0.5 SD between SARS-CoV-2 exposed versus unexposed infants.

© 2023 Firestein MR et al*. JAMA Network Open*.

eResults. Association of Enrollment Site With DAYC-2 Scores at Ages 6 to 11 Months

Unadjusted pairwise contrasts revealed DAYC-2 cognitive (β = 4.44, 95% CI = 0.93, 7.96), fine motor (β = 1.58, 95% CI = 0.45, 2.72), and expressive language (β = 3.77, 95% CI = 0.72, 6.83) scores were higher at the Utah site as compared to the Columbia site (eTable 3). Expressive language scores were also higher at the Alabama site compared to the Columbia site (β = 4.93, 95% CI = -0.39, 10.24) (eTable 3). However, in adjusted models there were no significant pairwise differences between enrollment sites and DAYC-2 cognitive, gross motor, fine motor, expressive language, or receptive language scores.

© 2023 Firestein MR et al*. JAMA Network Open*.

eFigure. Modified DAYC-2 Assessment Conducted Through a Video Visit

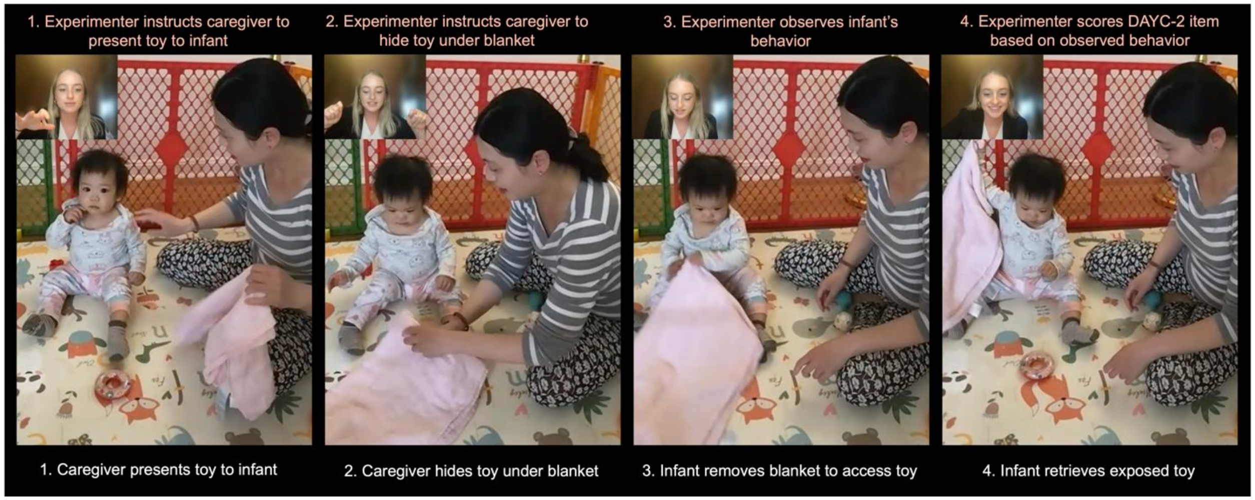

The experimenter, who is not visible to the mother and infant, but who can be heard by both, provides verbal instruction to the mother and directly observes the infant's response to each item. Permission to use videos and images was obtained.

© 2023 Firestein MR et al*. JAMA Network Open*.

eTable 1. Cause of Missing Scores in Each DAYC-2 Subdomain

	Cognitive	Receptive Language	Expressive Language	Gross Motor	Fine Motor
**Assessment Administration Issues**	11	7	5	13	4
**Infant Fussiness**	2	0	1	3	3
**Total**	13	7	6	16	7

Some infants included in this analysis did not complete all of the administered subdomains of the neurobehavioral assessment due to 1) technical issues with Zoom and/or internet connection, 2) infant fussiness, or 3) erroneous administration of the assessment that resulted in our inability to determine a ceiling, or test-terminating score, for the infant.

© 2023 Firestein MR et al. *JAMA Network Open*.

eTable 2. Demographic and Clinical Characteristics of Study Sample by Site

Variable	Full sample	CUIMC	UU	UAB	CUIMC (Reference) vs UU[b]		CUIMC (Reference) vs. UAB[b]		UU (Reference) vs. UAB[b]	
					p	Mean difference or odds ratio (95% confidence intervals)[a]	*p*	Mean difference or odds ratio (95% confidence intervals)	*p*	Mean difference or odds ratio (95% confidence intervals)
**Maternal Characteristics**	**(n=403)**	**(n=202)**	**(n=166)**	**(n=35)**						
**Age at Delivery (years)**					0.06	-1.08 (-2.19, 0.04)[c]	0.89	-0.15 (-2.15, 1.86)[c]	0.32	0.92 (-0.91, 2.77)[c]
Mean (SD)	32.1 (5.38)	32.6 (5.69)	31.5 (5.10)	32.4 (4.68)						
Median [Min, Max]	32.0 [18.7, 46.0]	32.9 [18.7, 46.0]	31.1 [20.4, 44.9]	32.4 [20.9, 43.3]						
**Maternal SARS-CoV-2 Infection Status**										
Unexposed	256 (63.5%)	106 (52.5%)	127 (76.5%)	23 (65.7%)	<0.001	2.95 (1.89,, 4.67)	0.55	1.74 (0.83, 3.78)	0.19	0.58 (0.27, 1.32)
Exposed during pregnancy	111 (27.5%)	73 (36.1%)	28 (16.9%)	10 (28.6%)	<0.001	0.36 (0.22, 0.58)	-0.35	0.71 (0.31, 1.51)	0.11	1.97 (0.82, 4.47)
Exposed pre-pregnancy/indeterminant	36 (8.9%)	23 (11.4%)	11 (6.6%)	2 (5.7%)	0.12	0.55 (0.25, 1.14)	0.32	0.47 (0.07, 1.7)	0.84	0.85 (0.12, 3.37)
**Primiparous**	182 (45.2%)	91 (45.0%)	78 (47.0%)	13 (37.1%)	0.71	1.08 (0.72, 1.63)	0.39	0.72 (0.33, 1.49)	0.29	0.66 (0.3, 1.39)
**Self-Reported Race**										
Asian or Asian American	13 (3.2%)	7 (3.5%)	6 (3.6%)	0 (0%)	0.94	1.04 (0.33, 3.21)	0.99	0 (0, 0)	0.99	N/A
Black or African American	45 (11.2%)	35 (17.3%)	0 (0%)	10 (28.6%)	0.98	N/A	0.22	1.67 (0.71, 3.68)	0.99	N/A
Native American or Alaskan Native	6 (1.5%)	4 (2.0%)	1 (0.6%)	1 (2.9%)	0.14	0.20 (0.01, 1.17)	0.97	0.96 (0.05, 5.86)	0.27	4.85 (0.18, 124.75)
Native Hawaiian or Other Pacific Islander	3 (0.7%)	2 (1.0%)	1 (0.6%)	0 (0%)	0.43	0.40 (0.02, 3.17)	1.00	N/A	1	N/A
White	240 (59.6%)	68 (33.7%)	149 (89.8%)	23 (65.7%)	<0.001	16.89 (9.69, 31.06)	<0.001	3.69 (1.76, 8.09)	<0.001	0.21 (0.09, 0.52)
Other or Mixed Race	45 (11.2%)	36 (17.8%)	9 (5.4%)	0 (0%)	<0.001	0.25 (0.11, 0.51)	1.00	#VALUE!	0.99	N/A

© 2023 Firestein MR et al. *JAMA Network Open*.

Declined or Unknown	51 (12.7%)	50 (24.8%)	0 (0%)	1 (2.9%)	0.99	N/A	0.21	0.26 (0.01, 1.35)	1	N/A
**Self-Reported Ethnicity**								0 (0, 0)		
Not Hispanic or Latino/a/x or Spanish	253 (62.8%)	71 (35.1%)	149 (89.8%)	33 (94.3%)	<0.001	16.17 (9.28, 29.70)	<0.001	30.44 (8.89, 191.17)	0.41	1.88 (0.5, 12.23)
Hispanic or Latino/a/x or Spanish	144 (35.7%)	125 (61.9%)	17 (10.2%)	2 (5.7%)	<0.001	0.07 (0.04, 0.12)	<0.001	0.03 (0, 0.12)	0.41	0.53 (0.08, 1.97)
Declined or Unknown	6 (1.5%)	6 (3.0%)	0 (0%)	0 (0%)	0.99	N/A	1.00	N/A	N/A	N/A
**Insurance status**										
Commercial Insurance	263 (65.3%)	78 (38.6%)	160 (96.4%)	25 (71.4%)	<0.001	42.39 (19.36, 111.95)	<0.001	3.97 (1.86, 9.09)	<0.001	0.09 (0.02, 0.27)
Medicaid Insurance	136 (33.7%)	121 (59.9%)	6 (3.6%)	9 (25.7%)	<0.001	0.03 (0.01, 0.05)	<0.001	0.23 (0.09, 0.5)	<0.001	9.23 (3.07, 29.63)
Insurance Status Unknown	4 (1.0%)	3 (1.5%)	0 (0%)	1 (2.9%)	0.99	N/A	0.57	1.95 (0.09, 15.74)	1.00	N/A
**Infant Characteristics**	**(n=204)**	**(n=167)**	**(n=36)**	**(n=407)**						
**Gestational Age at Birth (weeks)**					0.28	0.20 (-0.16, 0.55)[c]	0.62	-0.16 (-0.78, 0.46)[c]		-0.35 (-0.99, 0.27)[c]
Mean (SD)	38.7 (1.74)	38.6 (1.73)	38.8 (1.72)	38.5 (1.92)						
Median [Min, Max]	39.1 [28.9, 41.6]	39.0 [30.1, 41.6]	39.1 [28.9, 41.1]	39.1 [31.9, 41.1]						
**Preterm Birth (<37 weeks)**	40 (9.8%)	25 (12.3%)	13 (7.8%)	2 (5.6%)	0.16	1.65 (0.83, 3.44)	0.25	2.37 (0.66, 15.18)	0.64	1.43 (0.37, 9.44)
**Female Sex**	195 (47.9%)	80 (39.2%)	94 (56.3%)	21 (58.3%)	0.001	2.00 (1.32, 3.03)	0.03	2.17 (1.06, 4.52)	0.82	1.08 (0.52, 2.28)
**Vaginal Delivery**	266 (65.4%)	124 (60.8%)	121 (72.5%)	21 (58.3%)	0.02	1.70 (1.10, 2.65)	0.78	0.9 (0.44, 1.88)	0.10	0.53 (0.25, 1.13)
**Twin**	9 (2.2%)	5 (2.5%)	2 (1.2%)	2 (5.6%)	0.39	0.48 (0.07, 2.27)	0.32	2.34 (0.32, 11.35)	0.12	4.85 (0.56, 41.6)
**Age at Assessment (in months)**					<0.001	-1.85 (-2.17, -1.52)[c]	<0.001	-1.57 (-2.11, -1.03)[c]	0.36	0.27 (-0.31, 0.85)[c]
Mean (SD)	8.04 (1.81)	8.94 (1.52)	7.09 (1.63)	7.36 (1.57)						
Median [Min, Max]	8.00 [5.00, 11.0]	9.00 [5.00, 11.0]	6.00 [5.00, 11.0]	7.00 [5.00, 11.0]						
**Assessed in Spanish**	66 (16.2%)	65 (31.9%)	0 (0%)	1 (2.8%)	0.98	cN/A	0.0064	0.06 (0, 0.29)	1.00	N/A

[a]Comparisons were performed using logistic regression for categorical variables, and linear regression for continuous variables. N/A for Odds Ratio indicates comparison that includes condition with sample size of zero. [b]CUIMC: Columbia University Medical Center; UU: University of Utah, UAB: University of Alabama. [c]Denotes analyses for which the mean difference is reported. Mean differences and 95% CI were reported for continuous data, while odds ratios and 95% CI were reported for categorical data.

© 2023 Firestein MR et al. *JAMA Network Open*.

eTable 3. Comparison of DAYC-2 Subdomain Scores Across Enrollment Sites

					CUIMC (Reference) vs. UU				CUIMC (Reference) vs. UAB				UU (Reference) vs. UAB			
					**Unadjusted Model**		**Adjusted Model**		**Unadjusted Model**		**Adjusted Model**		**Unadjusted Model**		**Adjusted Model**	
	**Full Sample (N=407)**	**CUIMC (N=204)**	**UU (N=167)**	**UAB (N=36)**	**p value**	**Mean Difference (95% confidence intervals)**	**p value**	**Unadjusted Beta Value (95% confidence intervals)**	**p value**	**Mean Difference (95% confidence intervals)**	**P value**	**Unadjusted Beta Value (95% confidence intervals)**	**P value**	**Mean Difference (95% confidence intervals)**	**P value**	**Unadjusted Beta Value (95% confidence intervals)**
**Cognitive**					0.003	4.45 (1.52, 7.34)	<0.001	-0.26 (-4.46, 3.94)	0.24	3.08 (-2.09, 8.25)	0.003	-0.37 (-6.12, 5.39)	0.60	-1.37 (-6.57, 3.83)	0.68	-1.45 (-7.90, 5.00)
Mean (SD)	115 (14.4)	113 (14.2)	117 (13.9)	116 (16.1)												
Missing	11 (2.7%)	9 (4.4%)	2 (1.2%)	0 (0%)												
**Gross Motor**					0.25	-1.10 (-2.99, 0.79)	0.17	-0.23 (-3.02, 2.56)	0.76	0.53 (-2.91, 3.98)	0.527	0.55 (-3.47, 4.57)	0.25	1.63 (-1.18, 4.45)	0.51	1.96 (-1.53, 5.45)
Mean (SD)	99.9 (8.96)	100 (10.0)	99.3 (7.89)	101 (7.16)												
Missing	10 (2.5%)	7 (3.4%)	3 (1.8%)	0 (0%)												
**Fine Motor**					0.002	1.58 (0.61, 2.55)	0.01	0.91 (-0.53, 2.35)	0.39	0.65 (-0.84, 2.13)	0.01	-0.14 (-1.81, 1.53)	0.31	-0.94 (-2.75, 0.88)	0.29	-0.35 (-2.58, 1.88)
Mean (SD)	98.3 (4.68)	97.6 (4.23)	99.2 (5.22)	98.3 (3.81)												
Missing	6 (1.5%)	3 (1.5%)	3 (1.8%)	0 (0%)												
**Expressive Language**					0.005	3.77 (1.17, 6.37)	0.54	3.81 (-0.06, 7.67)	0.04	4.93 (0.28, 9.57)	0.72	4,04 (-1.44, 9.52)	0.60	1.15 (-3.16, 5.46)	0.69	3.24 (-2.12, 8.60)
Mean (SD)	102 (12.6)	99.8 (13.2)	104 (11.9)	105 (11.2)												
Missing	5 (1.2%)	1 (0.5%)	3 (1.8%)	1 (2.8%)												
**Receptive Language**					0.50	-0.78 (-3.04, 1.47)	0.05	11 (-2.28, 4.29)	0.21	2.62 (-1.53, 6.78)	0.05	3.20 (-1.50, 7.90)	0.07	3.41 (-0.23, 7.04)	0.03	1.34 (-2.96, 5.63)
Mean (SD)	101 (10.9)	101 (11.7)	99.9 (9.83)	103 (10.2)												
Missing	6 (1.5%)	3 (1.5%)	2 (1.2%)	1 (2.8%)												

CUIMC: Columbia University Medical Center; UU: University of Utah, UAB: University of Alabama

© 2023 Firestein MR et al. *JAMA Network Open*.

eTable 4. Comparison of DAYC-2 Subdomain Scores Between SARS-CoV-2 Nonexposure, First Trimester Exposure, Second Trimester Exposure, and Third Trimester Exposure

DAYC-2	Full Sample	SARS-CoV-2 Unexposed	SARS-CoV-2 Exposed			SARS-CoV-2 Unexposed vs. First Trimester Exposure				SARS-CoV-2 Unexposed vs. Second Trimester Exposure				SARS-CoV-2 Unexposed vs. Third Trimester Exposure			
						Unadjusted		Adjusted[1]		Unadjusted		Adjusted[1]		Unadjusted		Adjusted[1]	
			First Trimester	Second Trimester	Third Trimester	Beta Coefficient (95% CI)	p	Beta Coefficient (95% CI)	p	Beta Coefficient (95% CI)	p	Beta Coefficient (95% CI)	p	Beta Coefficient (95% CI)	p	Beta Coefficient (95% CI)	p
**Mean Score (SD)**	**(n=407)**	**(n=258)**	**(n=14)**	**(n=41)**	**(n=36)**												
Cognitive	115.1 (14.37)	115.7 (14.08)	116.4 (12.72)	111.4 (15.08)	115.1 (15.45)	0.73 (-6.85, 8.31)	0.85	2.25 (-5.84, 10.33)	0.59	-4.30 (-9.06, 0.47)	0.08	-1.42 (-6.53, 3.69)	0.59	-0.64 (-5.70, 4.42)	0.80	-1.54 (-6.52, 3.44)	0.54
Gross Motor	100.0 (8.96)	99.6 (9.16)	100.4 (7.07)	101.6 (8.72)	97.6 (9.32)	0.79 (-4.12, 5.69)	0.75	0.33 (-4.97, 5.64)	0.90	2.01 (-1.00, 5.03)	0.19	2.72 (-0.61, 6.05)	0.11	-2.00 (-5.34, 1.35)	0.24	-2.64 (-6.01, 0.73)	0.12
Fine Motor	98.3 (4.68)	98.4 (4.73)	99.8 (3.89)	97.7 (5.45)	98.9 (4.29)	1.39 (-1.14, 3.93)	0.28	1.23 (-1.51, 3.96)	0.38	-0.69 (-2.31, 0.93)	0.40	0.02 (-1.77, 1.80)	1.00	0.52 (-1.16, 2.21)	0.54	0.27 (-1.43, 1.97)	0.75
Expressive Language	101.8 (12.61)	102.0 (12.19)	100 (17.0)	101 (13.3)	98.7 (15.1)	-1.95 (-8.69, 4.79)	0.57	-1.22 (-8.65, 6.21)	0.75	-1.29 (-5.38, 2.80)	0.54	0.10 (-4.52, 4.73)	0.97	-3.23 (-7.69, 1.23)	0.15	-3.3 (-7.91, 1.24)	0.15
Receptive Language	100.6(10.86)	100.1 (10.19)	106 (9.41)	97.4 (14.4)	101 (12.2)	5.52 (0.03, 11.01)	0.05	5.20 (-0.77, 11.17)	0.09	-2.72 (-6.35, 0.91)	0.14	-2.81 (-6.79, 1.18)	0.17	1.10 (-2.56, 4.77)	0.55	-0.77 (-4.41, 2.86)	0.68

[1]Adjusted model includes race, ethnicity, maternal age at birth, insurance status, parity, delivery method, gestational age at birth, baby's sex, site, and language of assessment.
Note: The exact timing of exposure to maternal infection could be determined for 91 of 112 (81%) infants exposed during pregnancy.

© 2023 Firestein MR et al. *JAMA Network Open*.

eTable 5. Comparison of DAYC-2 Subdomain Scores Between SARS-CoV-2 Negativity During Pregnancy, Asymptomatic Infection During Pregnancy, and Symptomatic Infection During Pregnancy[1]

					**SARS-CoV-2 Unexposed vs. Asymptomatic Exposure**				**SARS-CoV-2 Unexposed vs. Symptomatic Exposure**			
					**Unadjusted Scores**		**Adjusted Scores[2]**		**Unadjusted Scores**		**Adjusted Scores[2]**	
**DAYC-2 Subdomain**	**Full Sample**	**SARS-CoV-2 Negative During Pregnancy**	**SARS-CoV-2 Asymptomatic Exposure**	**SARS-CoV-2 Symptomatic Exposure**	**Beta Coefficient (95% CI)**	***p***	**Beta Coefficient (95% CI)**	***p***	**Beta Coefficient (95% CI)**	***p***	**Beta Coefficient (95% CI)**	***p***
**Mean Score (SD)**	**(n=407)**	**(n=258)**	**(n=27)**	**(n=79)**								
Cognitive	115.1 (14.37)	115.7 (14.09)	117.0 (12.55)	112.7 (15.21)	1.30 (-4.35, 6.96)	0.65	5.44 (-0.42, 11.29)	0.07	-3.02 (-6.70, 0.66)	0.11	-1.99 (-5.76, 1.79)	0.30
Gross Motor	100.0 (8.96)	99.6 (9.16)	104.1 (5.72)	99.6 (9.36)	4.54 (0.93, 8.15)	0.01	4.43 (0.61, 8.26)	0.02	0.06 (-2.31, 2.43)	0.96	0.04 (-2.48, 2.56)	0.98
Fine Motor	98.3 (4.68)	98.4 (4.73)	98.1 (3.93)	98.5 (4.79)	-0.24 (-2.10, 1.62)	0.80	0.47 (-1.50, 2.43)	0.64	0.14 (-1.08, 1.36)	0.82	0.54 (-0.75, 1.82)	0.41
Expressive Language	101.8 (12.61)	102.0 (12.20)	102.8 (9.87)	99.1 (14.58)	0.87 (-3.91, 5.64)	0.72	1.74 (-3.40, 6.88)	0.51	-2.83 (-6.09, 0.42)	0.09	-1.84 (-5.35, 1.66)	0.30
Receptive Language	100.6(10.86)	100.1 (10.19)	102.52 (11.50)	100.4 (13.09)	2.40 (-1.71, 6.51)	0.25	2.64 (-1.73, 7.02)	0.24	0.25 (-2.53, 3.04)	0.86	-0.19 (-3.08, 2.71)	0.90

[1]This analysis only includes mothers who were confirmed SARS-CoV-2 negative during pregnancy and confirmed SARS-CoV-2 positive during pregnancy. Mothers with a pre-pregnancy/indeterminant SARS-CoV-2 infection were excluded (n=37). [2]Adjusted model includes race, ethnicity, maternal age at birth, insurance status, parity, delivery method, gestational age at birth, baby's sex, site, and language of assessment.

© 2023 Firestein MR et al. *JAMA Network Open*.

eReferences

1. Shuffrey, L. C. *et al.* Association of Birth During the COVID-19 Pandemic With Neurodevelopmental Status at 6 Months in Infants With and Without In Utero Exposure to Maternal SARS-CoV-2 Infection. *JAMA Pediatrics* e215563 (2022) doi:10.1001/jamapediatrics.2021.5563.
2. Saiman, L. *et al.* Infection prevention and control for labor and delivery, well baby nurseries, and neonatal intensive care units. *Semin Perinatol* **44**, 151320 (2020).
3. Dawood, F. S. *et al.* Incidence and Clinical Characteristics of and Risk Factors for Severe Acute Respiratory Syndrome Coronavirus 2 (SARS-CoV-2) Infection Among Pregnant Individuals in the United States. *Clinical Infectious Diseases* **74**, 2218–2226 (2022).
4. Metz, T. D. Is It Exposure to the Pandemic or to Maternal SARS-CoV-2 Infection That Is Adversely Affecting Early Childhood Neurodevelopment? *JAMA Network Open* **5**, e2215793 (2022).
5. Sutton, D., Fuchs, K., D'Alton, M. & Goffman, D. Universal Screening for SARS-CoV-2 in Women Admitted for Delivery. *New England Journal of Medicine* **382**, 2163–2164 (2020).

© 2023 Firestein MR et al. *JAMA Network Open*.
